# Bovine sperm HSP-70 molecules: a potential cryo-tolerance marker associated with semen quality and fertility rate

**DOI:** 10.3389/fvets.2023.1167594

**Published:** 2023-08-09

**Authors:** Berlin Pandapotan Pardede, Asmarani Kusumawati, Mulyoto Pangestu, Bambang Purwantara

**Affiliations:** ^1^Division of Reproduction and Obstetrics, School of Veterinary Medicine and Biomedical Sciences, IPB University, Bogor, Indonesia; ^2^Department of Reproduction, Obstetrics, and Gynecology, Faculty of Veterinary Medicine, Universitas Gadjah Mada, Yogyakarta, Indonesia; ^3^Department of Obstetrics and Gynecology, School of Clinical Sciences, Faculty of Medicine, Nursing and Health Sciences, Monash University, Victoria, VIC, Australia

**Keywords:** bull fertility, cryo-tolerance marker, freezability, HSP-70, sperm quality

## Abstract

**Introduction:**

Freezability is the ability of sperm to maintain its vitality and quality from various stress during the cryopreservation process, which is very important for the success of fertilization in AI programs. Heat shock proteins (HSPs) are unique proteins induced in response to various stress, including excess reactive oxygen species (ROS) and oxidative damage to intracellular enzymes that can harm cells. This study aimed to analyze the potential of HSP-70 molecules in bovine sperm as a marker of freezability or cryo-tolerance, as well as its association with semen quality and fertility rate.

**Methods:**

The classification of bulls is based on freezability (good freezability/GF and poor freezability/PF), which is obtained from the value of post-thaw viability using the SYBR-14/PI-flow cytometry. Semen quality assessed included sperm motility and kinetics (computer-assisted sperm analyses), plasma membrane integrity (HOS test), acrosome integrity (FITC-PNA), mitochondrial membrane (JC-1), and DNA damage (Halomax kit). The bull fertility rate assessment was analyzed based on the first service conception rate of each bull derived from data on the success of artificial insemination contained in the Indonesian-integrated National Animal Health Information System (iSIKHNAS). Gene expression levels of HSP-70 bovine sperm were performed using the RT-qPCR method. The protein abundance of HSP-70 bovine sperm was determined using the enzyme immunoassay (EIA) method.

**Results:**

Bovine sperm HSP-70 molecules, at the gene and protein level, showed a higher abundance in GF (*p* < 0.05) than in PF bulls. The percentage of each parameter of frozen–thawed sperm quality was significantly higher in GF (*p* < 0.05) than in PF bulls. The HSP-70 molecules at the gene and protein levels were significantly positively correlated (*p* < 0.01) with the fertility rate. Furthermore, HSP-70 molecules were negatively associated (*p* < 0.01) with low mitochondrial membrane potential and sperm DNA damage and positively correlated (*p* < 0.01) with other frozen–thawed sperm quality parameters. The overall quality of frozen–thawed sperm was closely related (*p* < 0.01) to the fertility rate.

**Conclusion:**

We may conclude that HSP-70 molecules in bovine sperm at the gene and protein level have the potential to be developed as a marker for cryo-tolerance or freezability, which may be utilized as a predictor of fertility and frozen–thawed sperm quality in bulls.

## Introduction

The fertility of the bulls is a crucial component in the evolution of the species (1). It is described as the capability of fertile sperm to penetrate oocytes to early embryonic development, and it is also an essential economic attribute in livestock genetic improvement (2). It is related to the need for various quality livestock products such as meat and milk globally and sustainably, especially in developing countries that cannot meet these needs independently. An artificial insemination (AI) program involving a genomic selection process using frozen sperm has been shown to accelerate the genetic improvement of livestock (3). The frozen sperm is produced through a series of processes called cryopreservation, a technique for storing or preserving sperm through a freezing process using low temperatures for future use (4). Freezability or cryo-ability of sperm is essential to the effectiveness of AI, which with specific advanced protocols and cryoprotectants, has been shown to increase the fertility of frozen–thawed sperm (5).

The several steps involved in cryopreservation, such as cooling, freezing, and thawing, harm sperm quality and cause dramatic alterations in sperm cells (4). The presence of intracellular ice crystal formation, osmotic injury, and structural and physiological damage to sperm cells due to oxidative stress was also reported due to the cryopreservation process (6). The decline in sperm quality due to oxidative stress, particularly damage to cell membranes, decreased viability and motility of up to 50%, and damage to the acrosome, ultimately impacts reduced fertility (4). It has also been observed that oxidative stress caused by an imbalance in reactive oxygen species (ROS) formation during the sperm cryopreservation procedure causes DNA fragmentation and affects male fertility (7). Furthermore, the sperm cryopreservation procedure increases free radical production due to cold shock and osmotic stress, which damages the semen plasma and leads to the death of sperm cells (4). Even though sperm freezability significantly affects male fertility and the economics of the livestock farming sector, there has been no standard approach that is ideal and accurate for assessing sperm freezability in a bull until now.

Cells and multicellular organisms respond to various forms of stress or other pressures by triggering or enhancing the production of a distinct protein family known as heat shock proteins or HSPs (8). Even though the precise role of HSPs is not yet known, it is known that they help protect cells from the harmful effects of a stressful and damaging environment (9). Many studies have shown that HSPs, especially the abundantly expressed 70 kDa HSP (HSP-70), play an essential role in anticipating heat tolerance, and HSP-70 has been reported to act as an indicator of heat tolerance in a cell (9). A decrease in HSPs was also reported to increase ROS concentrations and oxidative damage to intracellular enzymes (10). HSP-70, one of the chaperone molecules usually induced during stress on cells, is reported to be distributed in spermatogonia, spermatid, and sperm cells, and its localization can change during spermatogenesis, after ejaculation, to the freezing process (11). In addition, the HSP-70 protein is considered an essential element for the process of meiosis and maturation of sperm, as well as preventing apoptosis because it can activate the natural immune system and play a role in immunomodulation (12). HSP-70 also plays a role in sperm-egg membrane interactions (13). In addition, HSP-70 can increase membrane fluidity as a protector for sperm in the female reproductive tract (14). Current findings on vertebrates suggest that HSP-70 plays a significant part in the response of multicellular animals to various stimuli; one of them, the protection of cells against the effects of heat stress, has been observed in lizards (15). Whether HSP-70 can function or not as an indicator of heat or cold tolerance, specifically caused by the cryopreservation process on livestock sperm cells, requires further study. The primary purpose of this research was to explore the assumption that sperm integrity and freezability are linked to cellular and functional processes in sperm. Furthermore, it specifically identifies the potential of the HSP-70 molecule at the gene and protein level as one of the molecules in sperm that controls tolerance to the cryopreservation process, which is associated with various parameters of semen quality and field fertility levels.

## Materials and methods

### Sperm freezability determination and experimental design

This study only used cryopreserved sperm, a commercial product from an AI center, as the primary sample and did not directly involve bulls. The cryopreserved sperm samples were produced from 20 bulls of the same productive age (4 to 6 years). Furthermore, the entire sperm collection process was carried out using an artificial vagina for the same period (without seasonal differences), frozen using an egg-yolk-tris-based extender. To eliminate any potential for variation in the samples, the bulls were kept in the same environment regarding feeding and handling management. However, each stage follows the operational standards in Indonesia, SNI ISO 9001: 2015 No. G.01-ID0139-VIII-2019, supervised by a veterinarian, considered the principles of animal welfare, which refer to the ethical clearance requirements of the Animal Care and Uses Committee. The approach previously reported by other studies was used to determine sperm freezability (16). In summary, the bulls used were determined regarding their post-thaw viability. Flow cytometry measured cell viability post-thawing (CyFlow SL, Partec, Germany). The percentages of viable and nonviable sperm were determined using an SYBR-14/PI, Live/Dead Sperm Viability Kit L-7011, Thermo Fisher Scientific double staining protocol. The percentage of viable sperm, as measured by green fluorescence, was used as a criterion for determining the bulls with good or poor semen freezability. After that, the post-thaw viability of the sperm from each bull used in the study was calculated as the average. The average value of post-thaw viability was 73.84%, ranging from 61.50 to 88.10%. Based on the average post-thaw viability values and the differences from the population average, the bulls were then categorized as bulls with good (GF) or poor semen freezability (PF). Our cutoff for driving a wedge into GF and PF categories is the mean of the entire population. Bulls with higher-than-average sperm viability values were classified as GF, whereas those with lower viability were classified as PF. Table 1 summarizes the classification of bulls used in this work based on post-thaw viability and freezability.

**Table 1:** Sperm freezability phenotypes of the bulls used for further analysis: Bulls 1–10 was defined as good freezability (GF), and Bulls 11–20 were grouped as poor freezability (PF).

Bull No.	Freezability status	Average post-thaw viability (%)	Difference from population average (%)
1	Good freezability	82.60	8.76
2		88.10	14.26
3		85.70	11.86
4		85.10	11.26
5		85.30	11.46
6		84.50	10.66
7		87.40	13.56
8		79.40	5.56
9		83.60	9.76
10		81.60	7.76
11	Poor freezability	61.60	−12.24
12		63.60	−10.34
13		61.50	−12.34
14		62.00	−11.84
15		64.00	−9.84
16		63.30	−10.54
17		66.00	−7.84
18		65.80	−8.04
19		63.60	−10.24
20		62.20	−11.64

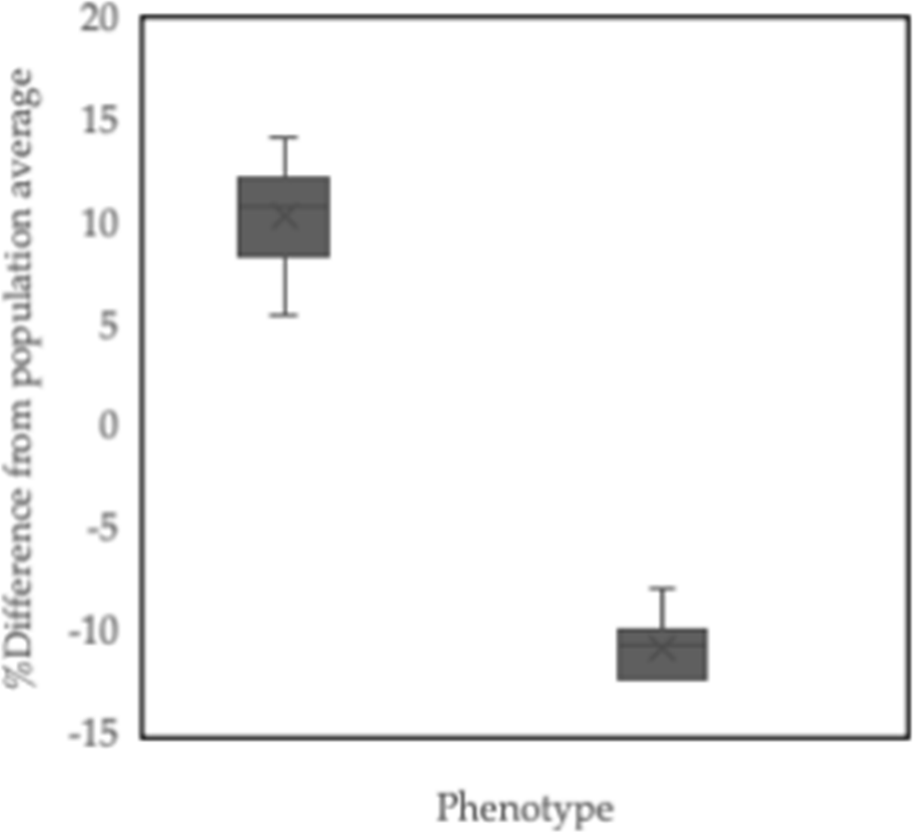
 Bulls were classified as good freezability and poor freezability based on average post-thaw viability scores and the percent differences from the population average (*p* < 0.05).

### Cryopreserved sperm quality assessment

The motility and kinetic characteristics of sperm cells were measured using computer-assisted sperm analyses (CASA). Sperm cells were thawed in a 37°C water bath for 30 s. Each sample was dispensed (5 μL) onto a preheated slide, and 250 sperm count in each slide was promptly measured. Four parameters were assessed, including progressive motility (PM), curvilinear velocity (VCL), straight-line velocity (VSL), and average path velocity (VAP). Evaluation of sperm plasma membrane integrity (PMI) was performed using the hypoosmotic swelling (HOS) test, which is based on the procedure developed by Pardede et al. (3). Briefly, cryopreserved sperm cells was thawed in a 37°C water bath for 30 s and mixed in the HOS test solution (0.735 gr Na citrate (Merck, Darmstadt, Germany), 1.351 gr fructose (Merck, Darmstadt, Germany), 100 mL of aquabidest), which was then incubated in a water bath at 37°C for 30 min. Each sample was plated out at a concentration of 5 μL. Using a phase-contrast microscope with a 40X objective, 250 sperm from each sample were counted and categorized as HOS-positive (coiled tail present) or HOS-negative (coiled tail absent). FITC-PNA and propidium iodide (PI)-based fluorescent staining (Sigma, St. Luis MO) was used to evaluate sperm acrosome integrity (SAI) according to the method of Rajabi-Toustani et al. (17). Cryopreserved sperm cells were thawed in a 37°C water bath for 30 s. Each sample was smeared into a slide (5 μL) before being air-dried at room temperature. Fixation of the smear sample in 96% ethanol for 10 min at room temperature and then air-dried. Drop 30 μL (100 μg/mL) Peanut agglutinin (PNA) lectin solution, which was then incubated at 37°C for 30 min. The preparations were then dripped with 5 μL (1 μg/μL) of PI (Sigma, St. Luis MO) and incubated for 5 min. The slides were incubated and rinsed in phosphate-buffered saline (PBS), and a coverslip was placed on top. SAI status was identified using a fluorescent microscope at 380–420 nm wavelengths. The number of sperm observed in each sample slide was 250 sperm cells. The evaluation results were divided into two categories, sperm with green fluorescent acrosomes were categorized as intact acrosomes, while sperm with red fluorescence were categorized as damaged acrosomes. All procedures were carried out in a dark room.

The mitochondrial membrane potential (MMP) was assessed using JC-1 fluorescence-based staining (Molecular Probe Inc., Eugene, United States), as described by Gloria et al. (18). Cryopreserved sperm cells were thawed in a 37°C water bath for 30 s. The sperm pellet from each semen sample was centrifuged for 15 min at room temperature and then washed in PBS (Sigma-Aldrich Chemie Gmbh, Steinheim, Germany). The sperm pellet from the semen sample was centrifuged twice, washed, and resuspended in 1 mL PBS. Samples were stained with 10 μg/mL JC-1 at 37°C for 15 min. After incubation, the sperm sample was evaluated by fluorescence microscopy. At least 250 sperm per sample were evaluated using suitable filters. Sperm show bright yellow/orange in the midpiece if they have high MMP and a green reaction if they have low MMP. Sperm Halomax Kit^®^ combined with a Wright’s eosin methylene blue solution was used to evaluate DNA damage, which refers to the method of Garcı ´a-Macı ´as et al. (19). Sperm cells were thawed in a 37°C water bath for 30 s. The sperm sample, which contained 15–20×10^6^ cells/ml, was added to 50 μL of liquid agarose before being mixed. The sperm sample is then dripped into the marked wells from the pre-cooled glass plate (4°C), covered, and transferred to the refrigerator at 4°C for 5 min. Then remove the slide from the fridge and carefully remove the coverslip at room temperature (22°C). Next, drip the lysate solution on the slide for 5 min and dry it by tilting it. Wash the slides for 5 min with distilled water and dry them by tilting them again. Then, the slides were dehydrated in ethanol solution (70 and 90%) for 2 min each, and dry the slides. Put the slide into Wright’s eosin methylene blue solution in the last step. Under a microscope, five hundred sperm cells were assessed. Sperm that have normal DNA show a small halo, in contrast to the sperm that contain damaged DNA, which shows a huge halo.

### Bull fertility rate assessment

The bull fertility assessment is determined by the value of the first service conception rate of each bull based on the success of AI. The data are in the form of an AI application using cryopreserved sperm straws from bulls used in the study and the results of pregnancy examinations for each inseminated female cow. More than 10,000 data on the success of AI over the last 2 years were used in the study to analyze the percentage of first service conception rate in each bull using the Pardede et al. (20) technique. The first service conception rate was defined as the number of pregnant cows (80–100 days after the first AI service) to all inseminated cows. However, the AI success data used is secondary data reported by AI officers in the field, which is contained and recorded in the Indonesian - integrated National Animal Health Information System (iSIKHNAS) and does not directly involve female cows. The results of the %fertility rate analysis for each bull sequentially were 80.91, 80.22, 80.44, 78.93, 78.52, 79.68, 78.93, 79.68, 79.26, 77.07, 53.89, 54.91, 57.56, 54.91, 52.95, 51.80, 58.25, 54.48, 56.08, and 54.89%. The analysis results were then used to predict the %fertility rate for each bull in the study.

### Bovine sperm HSP-70 molecules abundance assessment

The abundance of HSP-70 molecules in bovine sperm was evaluated based on the level of gene expression and the amount of protein found in the sample, as described by Pardede et al. (21). Gene expression levels of HSP-70 bovine sperm was performed using the RT-qPCR method. For 30 s, sperm cells were thawed in a water bath maintained at 37°C. After thawing, the sperm was centrifuged at 16,000 g for 15 min and rinsed three times with PBS. Following the manufacturer’s instructions, total RNA was extracted using the TRI reagent (Zymo Research, USA). To sum up, the sperm pellet was resuspended in 1 mL TRI reagent by repeated pipetting. Close the sample container tightly after adding 0.1 mL of BCP or 0.2 mL of chloroform for every 1 mL of TRI reagent. Then the sample was centrifuged at 12,000 g for 15 min at 4°C and allowed to stand for 5–10 min. The sample was centrifuged at 12,000 g for 8 min at 25°C until an RNA precipitate formed. Next, remove the supernatant, wash the RNA pellet using 75% ethanol, and centrifuge at 7,500 g for 5 min at 25°C. After 3–5 min, discard the ethanol wash and let the RNA pellet air dry. Using a NanoDropTM One/OneC Microvolume UV–Vis Spectrophotometer, quantify and characterize total RNA (Thermo Scientific). The SensiFASTTM cDNA Synthesis Kit (Bioline^®^ Ltd., United Kingdom, Bio-65,054) synthesized cDNA following the manufacturer’s instructions. After this process, as little as 20 μL of cDNA is needed for RT-qPCR. NanoDropTM One/OneC Microvolume UV–Vis Spectrophotometer was used for quantifying total cDNA and analyzing its purity (Thermo Scientific). Transcript abundance was measured using quantitative real-time polymerase chain reaction (qPCR). SsoFastTM EvaGreen^®^ Supermix was used for the qPCR reactions (Bio-Rad Lab, California, United States). Twenty microliters of a qPCR reaction containing 10 μL SsoFast EvaGreen Supermix, 1 μL of forward (F) and reverse (R) primers, 2 μL of reverse cDNA, and 6 μL of water-free DNase/RNase were employed. The genes found in this investigation were housekeeping gene PPIA (XM 001252921.1; forward: 5’-ATGCTGGCCCCAACACAA-3′ and reverse: 5′- CCCTTTCACCTTGCCAAA −3′) and HSP-70 (HSP-70; NM 174344.1; forward: 5’-TTGGGGACAAGTCAGAGAATG-3′ and reverse: 5’-ATCGTGGTGTTCCTTTTGATG-3′). Expression levels of the HSP-70 gene were quantified using the 2-ΔΔCt method, which compares the CT value of the target gene to that of PPIA.

The protein abundance of HSP-70 bovine sperm was determined using the enzyme immunoassay (EIA) method. For 30 s, sperm cells were thawed in a water bath maintained at 37°C. The sperm sample was washed twice with PBS and centrifuged at 12,000 g for 15 min. The protein abundance of HSP-70 bovine sperm was determined using EIA on the sperm pellet per the package guidelines (Cat. No. MBS7606199, MyBioSource.com). Once everything was ready, 100 μL of diluted sperm sample was added to each well, and the plates were incubated at 37°C for 90 min with the covers on. After incubating at 37°C for 60 min, the plate should be washed twice with wash buffer solution, 100 μL of biotin-labeled antibody working solution should be added to each well, and the plate should be covered once more. Three more times, wash the plate using the wash buffer solution, leaving the solution in the wells for 1–2 min. Then, place 100 μL of the HRP-streptavidin conjugate working solution in each well, cover the plate, and incubate it at 37°C for another half an hour. Then, wash the plate 5 times with wash buffer solution, letting each time’s wash buffer sit in the wells for 1–2 min. Cover the plate again, incubate at 37°C in the dark for 10–20 min, and then add 90 μL of TMB substrate to each well. Subsequently, 50 μL of stop solution is added to each well, and the resulting color reaction is observed. At a wavelength of 450 nm, an EIA reader will be used to determine the absorbance value. By comparing the value of the sample to the standard curve, the concentration of HSP-70 protein in the sample may be calculated.

### Statistical analysis

Twenty breeding bulls (*n* = 10 Good, *n* = 10 Poor) were utilized for the statistical test. The data with a freezability trait were analyzed using a generalized linear mixed model. Data analysis to determine the grouping of breeding bulls based on the freezability phenotype has been explained in the previous subsection. The t-test was used to analyze the cryopreserved sperm quality, %fertility rate, and abundance of bovine sperm HSP-70 molecules. The results of the study are shown as a mean ± standard error. The correlation between the various variables was analyzed using Pearson’s test. Using a Scatter Plot linearity test, we identified a pattern of association between gene expression, HSP-70 protein abundance, and % fertility. SPSS 25.0 was used to analyze the data.

## Results

**Figure 1:**
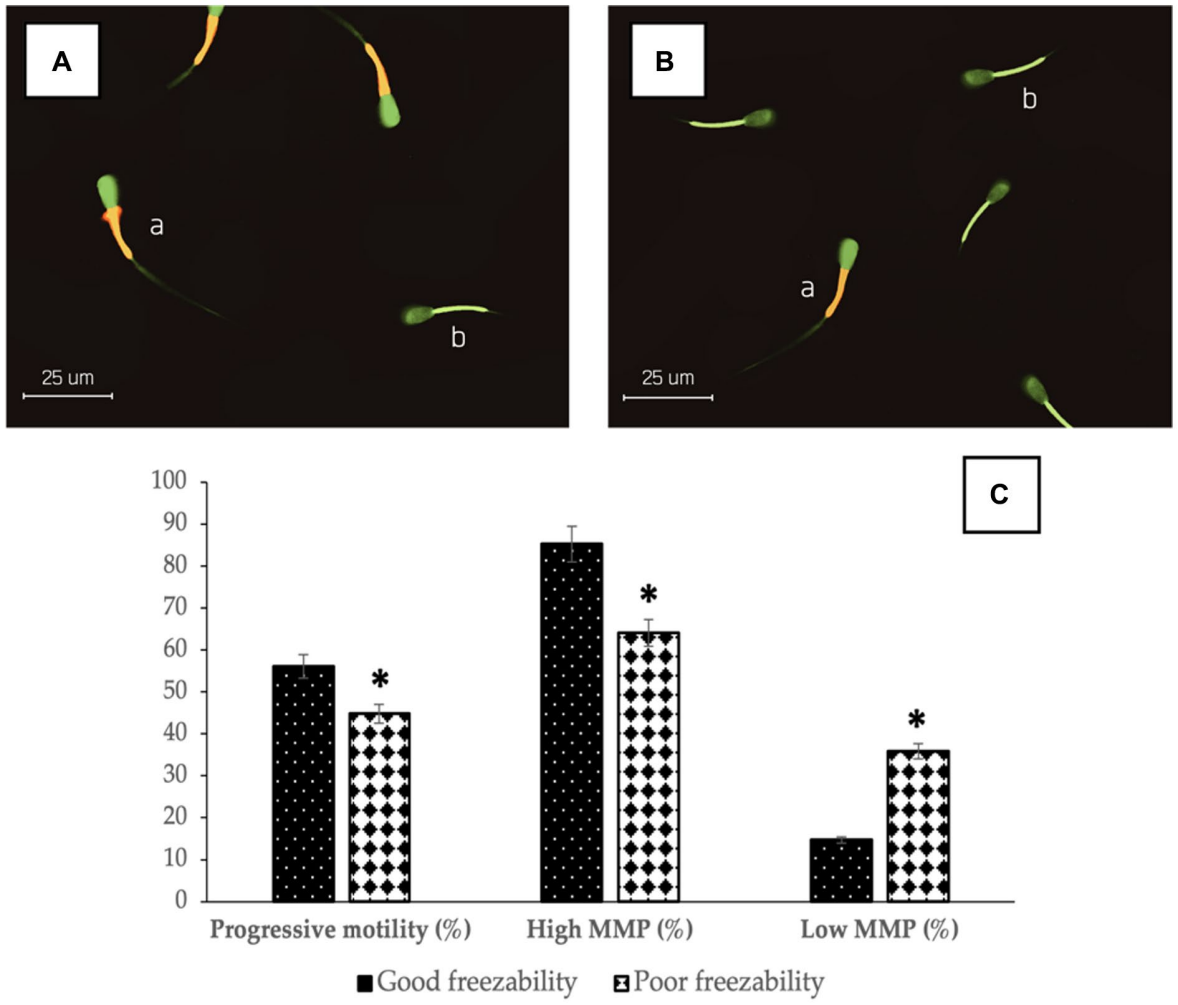
The photomicrograph of sperm in JC-1 fluorescence-based staining of GF **(A)** and PF **(B)** bulls; Sperm show bright yellow/orange in the midpiece if they have high MMP (a) and a green reaction if they have low MMP (b). The percentage of progressive motility and mitochondrial membrane status (high and low) in bovine sperm **(C)** with different freezability groups (GF vs. PF). *Significant difference when compared to PF (*p* < 0.05).

The results showed that there was a significant difference (*p* < 0.05) in %PM (56.14 ± 0.35% vs. 44.83 ± 0.31%) (Figure 1) and %sperm velocity (Table 2) between the GF and PF groups. These results were also supported by the results of potential mitochondrial membrane analysis, High MMP (85.31 ± 0.37% vs. 64.09 ± 0.34%) and Low MMP (14.69 ± 0.37% vs. 35.91 ± 0.34%), which also showed a significant difference (*p* < 0.05) between the freezability groups (Figure 1). Likewise, with %PMI, membrane integrity in the GF group was higher (*p* < 0.05) compared to the PF group (Table 2).

**Table 2:** The difference in the sperm parameters and fertility rate, with freezability groups, GF vs. PF bulls.

Parameters	Good freezability*	Poor freezability
VCL (μm/s)	134.69 ± 0.39	112.91 ± 1.48
VAP (μm/s)	85.24 ± 0.44	64.33 ± 0.81
VSL (μm/s)	54.82 ± 0.44	44.40 ± 0.40
PMI (%)	84.43 ± 0.52	64.05 ± 0.37
% Fertility rate	79.36 ± 0.14	54.97 ± 0.26

VCL: curvilinear velocity; VAP: average path velocity; VSL: straight line velocity; PMI: Plasma membrane integrity. ^*^Significant difference when compared to LF (*p* < 0.05).

**Figure 2:**
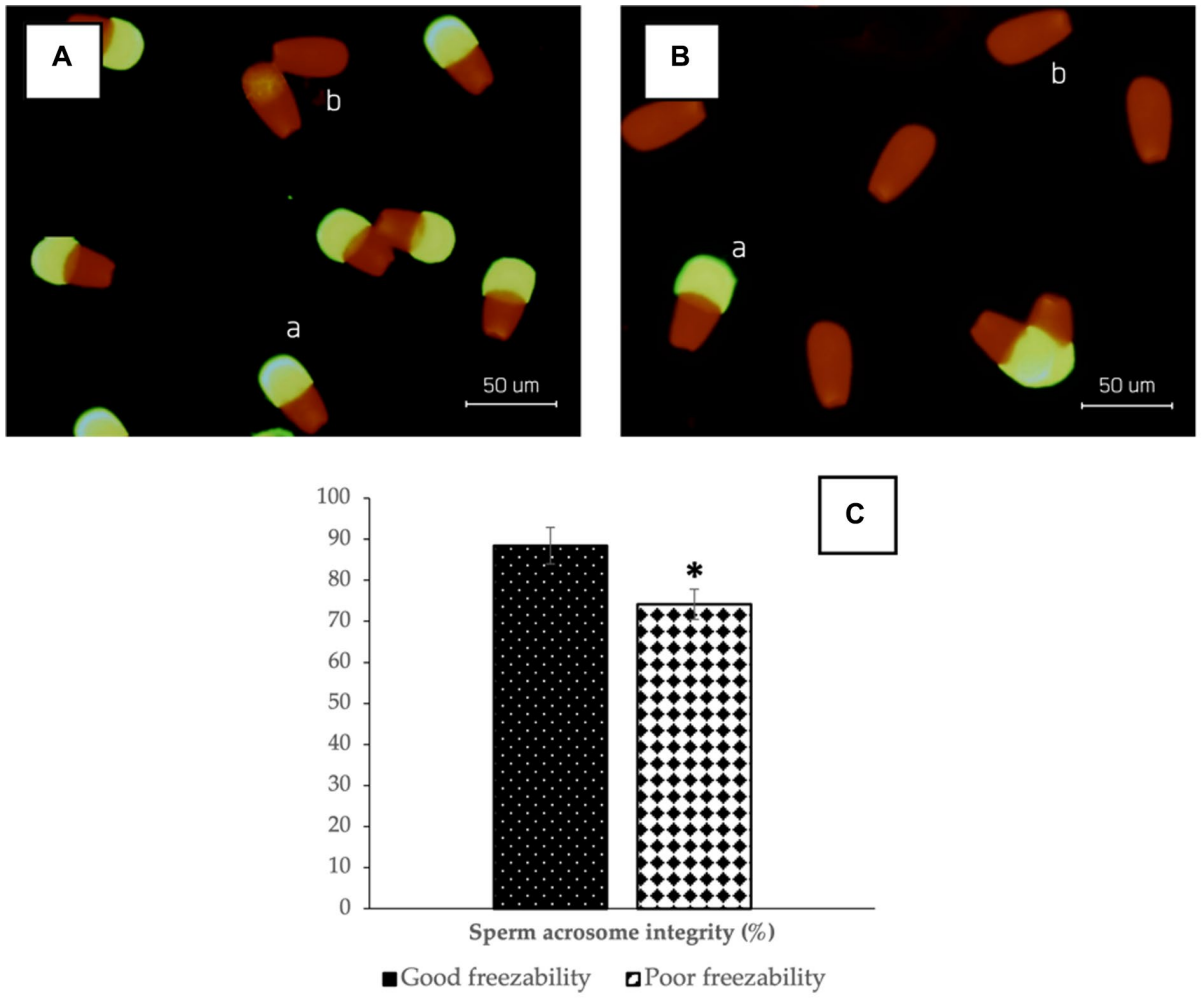
The photomicrograph of sperm in FITC-PNA and propidium iodide (PI)-based fluorescent staining of GF **(A)** and PF **(B)** bulls; Sperm with green fluorescent acrosomes were categorized as intact acrosomes (a), while sperm with red fluorescence were categorized as damaged acrosomes (b). The percentage of sperm acrosome integrity in bovine sperm **(C)** with different freezability groups (GF vs. PF). *Significant difference when compared to PF (*p* < 0.05).

**Figure 3:**
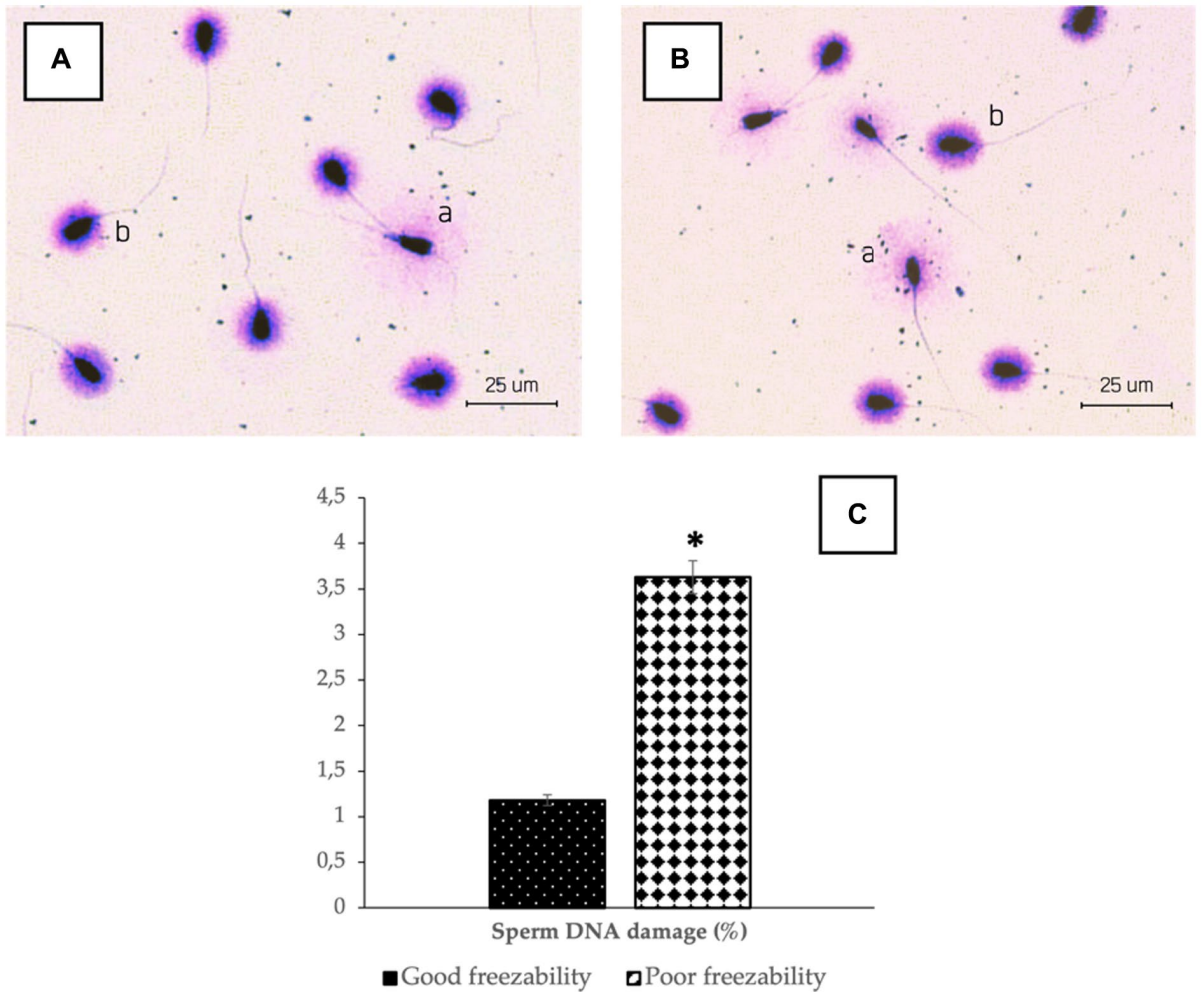
The photomicrograph of sperm in Halomax Kit combined with a Wright’s eosin methylene blue staining of GF **(A)** and PF **(B)** bulls; Sperm that have normal DNA show a small halo (b), in contrast to the sperm that contain damaged DNA, which shows a huge halo (a). The percentage of sperm DNA damage in bovine sperm **(C)** with different freezability groups (GF vs. PF). *Significant difference when compared to PF (*p* < 0.05).

The results of sperm acrosome integrity analysis in the two freezability groups in this study also showed a difference, in which the %SAI in the GF group (88.41 ± 0.60%) was higher (*p* < 0.05) compared to the PF group (74.16 ± 0.34%) (Figure 2). Sperm DNA damage was also found to be higher in the PF group (*p* < 0.05) than in the GF group (Figure 3). The low freezability of bulls proven to have a low fertility rate was also found in this study (Table 2). Furthermore, this study strengthens the hypothesis so far that the overall quality of frozen–thawed sperm is closely related (*p* < 0.01) and affects the level of fertility produced in the field (Table 3).

**Table 3:** Correlation between HSP-70 gene expression and protein abundance, semen quality parameters, and fertility rate in the bulls.^a^

Parameters	H-gene	H-protein	PM	VCL	VAP	VSL	PMI	H-MMP	L-MMP	SAI	DNA-d	%Fertility
H-gene	1	0.872	0.865	0.730	0.830	0.787	0.851	0.895	−0.895	0.862	−0.901	0.913
H-protein		1	0.887	0.782	0.866	0.817	0.879	0.909	−0.909	0.838	−0.952	0.932
PM			1	0.749	0.848	0.799	0.843	0.908	−0.908	0.830	−0.890	0.910
VCL				1	0.887	0.721	0.764	0.792	−0.792	0.756	−0.780	0.820
VAP					1	0.791	0.845	0.886	−0.886	0.841	−0.887	0.909
VSL						1	0.786	0.839	−0.839	0.768	−0.802	0.861
PMI							1	0.882	−0.882	0.812	−0.878	0.903
H-MMP								1	−1.000	0.874	−0.917	0.964
L-MMP									1	−0.874	0.917	−0.964
SAI										1	−0.857	0.887
DNA-d											1	−0.948
%Fertility												1

H-gene, HSP-70 gene expression; H-protein, HSP-70 protein abundance; PM, progressive motility; VCL, curvilinear velocity; VAP, average path velocity; VSL, straight line velocity; PMI, plasma membrane integrity; H-MMP, high mitochondrial membrane potential; L-MMP, low mitochondrial membrane potential; SAI, sperm acrosome integrity; DNA-d, sperm DNA damage. ^a^The Pearson correlation represents all values obtained, regardless of the grouping of bulls based on freezability. All the tested variables correlated significantly at the 0.01 level.

**Figure 4:**
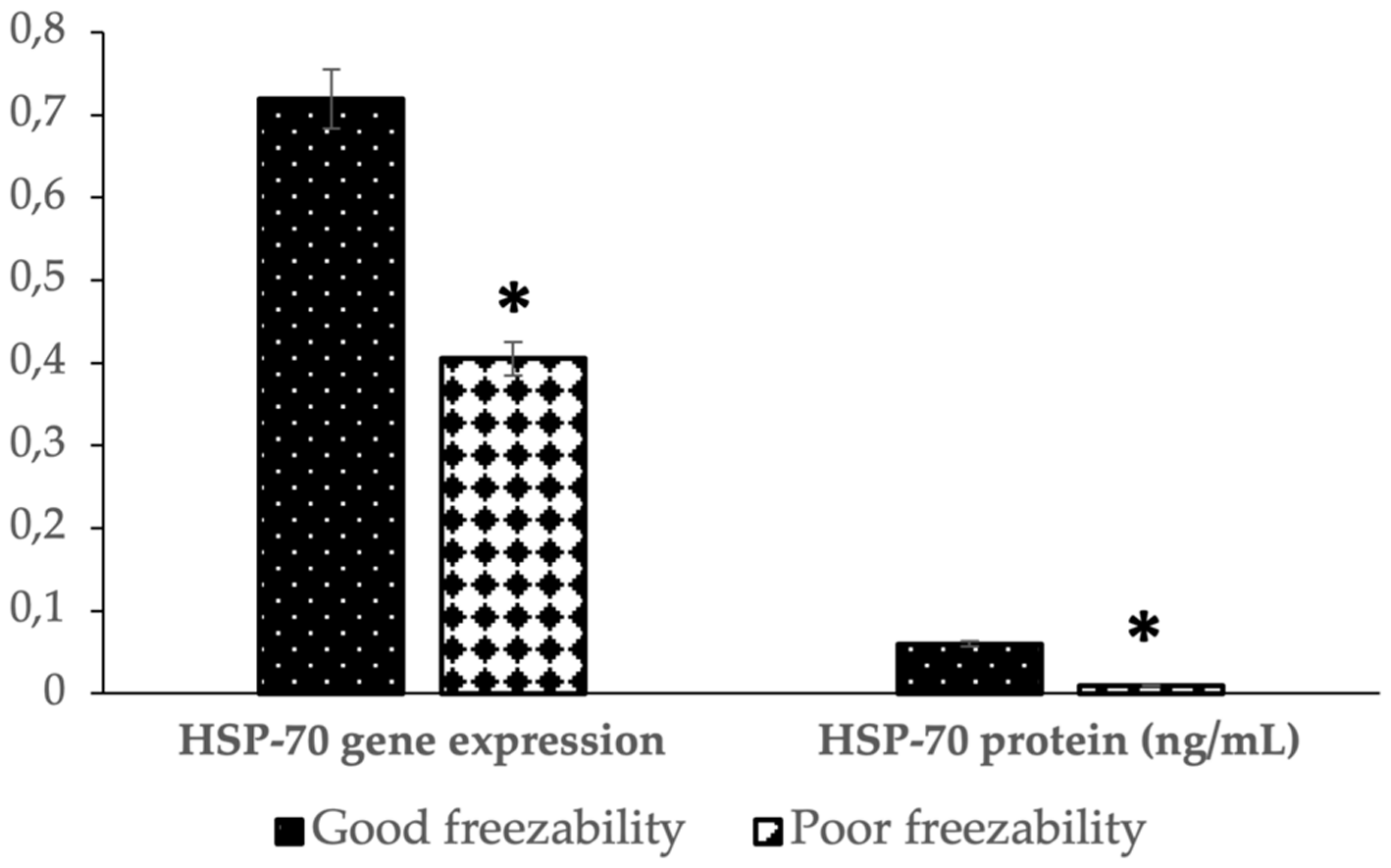
The HSP-70 gene expression and protein abundance in bovine sperm with different freezability groups (GF vs. PF). ^*^Significant difference when compared to PF (*p* < 0.05).

**Figure 5:**
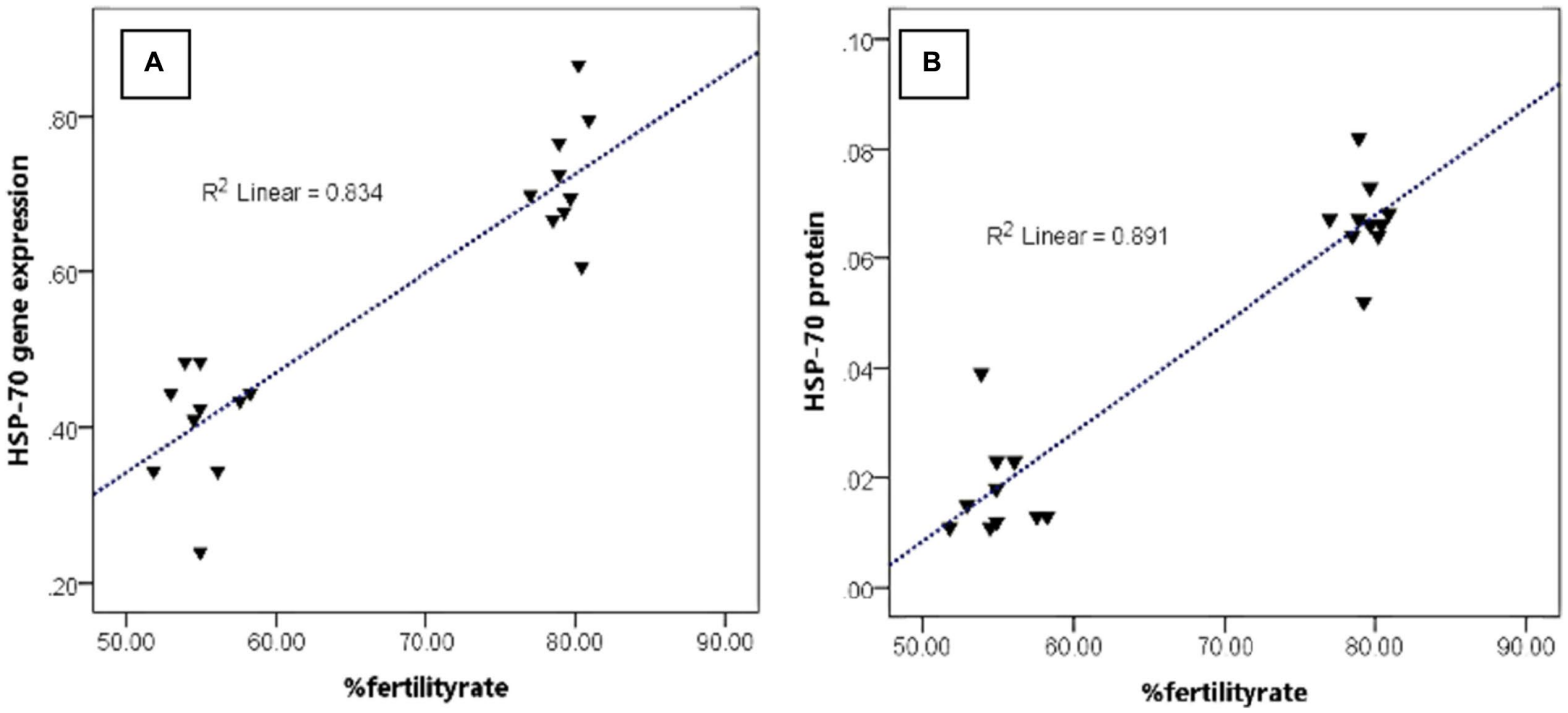
The relationship pattern between the HSP-70 gene and protein abundance with %fertility rate regardless of bull grouping based on freezability.

Figure 4 shows that the abundance of bovine sperm HSP-70 molecules at the gene and protein level showed a higher abundance (*p* < 0.05) in bulls with high freezability than in low freezability. Significantly (*p* < 0.01), both were closely related to the level of fertility in the field (Table 3), with a high level of linearity, HSP-70 gene (R2:0.834) and protein (R2:0.891) (Figure 5).

## Discussion

HSPs are a family of molecules that work together to help cells deal with stress and continue functioning normally (22–24). In eukaryotic cells, tolerance is shaped by the degree to which HSPs are expressed (8). Numerous reports have shown that cellular HSPs expression levels shift in response to thermal and cold stress (24). This study also found these findings; even the HSP-70 molecule was associated with sperm movement patterns. As it is known that the pattern of sperm movement is one of the essential parameters that ensure that cells will reach the site of fertilization. On the other hand, Casas et al. (25) stated that HSPs are one of the vitally important factors for sperm fertilization ability. HSP-70 was also reported to be low expressed in infertile men (26). The findings in this study also report that the abundance of HSP-70 molecules is closely related to the fertility rate. These things indicate that the HSP-70 molecule can affect the level of freezability, movement patterns of sperm, and sperm’s ability to fertilize.

Although HSP-70 has been considered a cytosolic protein, the actual mechanism of sperm function is still unclear. According to research by Webster et al. (27), HSPs can inhibit the stress-induced aggregation of unfolded proteins. Non-native folding intermediates can be helped along to their native state by the HSP-70 chaperones system (28). This study’s findings of decreased HSP-70 gene expression and protein abundance in PF bulls are likely a reaction to the degradation or consumption of chaperonins, which are typically present in large quantities and keep protein shape stable during cryopreservation. Low levels of gene expression and the amount of HSP-70 protein may contribute to the reduced membrane plasma integrity seen in the PF bulls. Since HSP-70 can form complexes with lipids, it has been postulated that it may play a role in the folding of membrane proteins and transposing polypeptides across membranes (29). Insufficient HSP-70 expression was found to be the cause of inadequate protein production. The proteins in the sperm membrane end up folding incorrectly as a result. The sperm membrane’s flexibility could be compromised. According to Aboagla and Terada (30), the membrane proteins are crucial for the fluidity of the sperm membrane. This finding suggests a correlation between sperm membrane rigidity and reduced motility. A lack of sperm motility, membrane integrity, and HSP-70 expression was found in PF bulls. These results indicate a possible link between low HSP-70 gene expression, protein abundance, and low sperm motility. However, further research is needed to determine how low or no HSP-70 expression affects sperm motility throughout the freezing process.

Possible causes of sperm quality decline include membrane damage sustained during freezing and thawing. Physical and chemical stress on the sperm plasma membrane during cryopreservation has been linked to oxidative stress and the development of excess free radical oxygen species (ROS), leading to sperm destruction (31). By modulating the activity of several enzymes, HSP-70 can control how cells perform their functions. We found that low levels of HSP-70 gene expression and protein abundance correlated with reduced sperm motility. It suggests that changes in gene expression levels and HSP-70 protein abundance led to decreased antioxidant enzyme activity in cells; relatively more ROS are formed and damage sperm, which in turn causes a reduction in sperm motility. In response to stress, cells activate a group of homologous stress-activated proteins (SAPs) that includes protein kinases; HSP-70 can inhibit the activity of these kinases, preventing cell death (24). Enhanced HSP-70 expression in cells inhibits the resistance-enhancing effects of protein kinases, including p38 and jun N-terminal kinase (JNK) (32).

According to our research findings, a positive association exists between the amount of gene expression, the abundance of HSP-70 protein, and sperm motility. The results of this research and earlier studies suggest that an increase in HSP-70 production can help sperm cells become more resistant to the damaging effects of cryopreservation. The HSP-70 molecule can restore membrane integrity, increase Ca2 + -ATPase function even when it has been suppressed by stress reactions, and lessen the negative impact of stress reactions on mitochondria (33). In the meantime, heat stress can cause a rise in superoxide dismutase (SOD) production, which can be prevented by the heat-shock protein HSP-70, shielding cells from oxidative damage. HSP-70 may regulate SOD activity to protect sperm membranes from the damaging effects of ROS (34). As cells are under pressure, they turn on a protein synthesis regulator called eukaryotic initiation factor 2 (EIF- α). It regulates the translation of specific stress-induced mRNAs and mediates a temporary decrease in global translation (35). Previous research demonstrated that cells would upregulate HSPs expression levels in response to stress (36). This finding supports the hypothesis that EIF-α dephosphorylates in response to cellular stress, allowing it to produce additional proteins necessary for the cell’s survival.

Nevertheless, the data showed that PF bulls had low levels of HSP-70 gene expression and protein abundance. The function of sperm cells may be impaired because they have been stressed beyond their capability. It has been demonstrated that HSPs have an intrinsic ATPase, which is necessary for activating quality factor proteins *in vivo* (37). Another finding suggests that the HSP-70 protein’s chaperone action depends on ATP hydrolysis (28). Sperm motility was strongly correlated with mitochondrial respiratory complex activity (38). Since sperm might lack the energy to keep their vigor steady, sperm motility may decline.

Furthermore, ATPase activity is maintained only in the optimal temperature range and is reduced in response to cold shock. Thus, a decrease in ATPase activity induces a reduction in sperm motility activity (38). The high mitochondrial membrane was lower in bulls with poor freezability and vice versa in this study. Chianese and Pierantoni (39) revealed that mitochondria are organelles in sperm cells that are the site for ATP production, which produces the energy needed for sperm to be able to move and motile until they reach the site of fertilization. Poor freezability indicates an excessive increase in oxidative stress and causes an increase in lipid peroxidation and the formation of ROS in cell mitochondria. Singh et al. (40) stated that oxygen in mitochondria plays a crucial role in oxidative phosphorylation, which involves the breakdown of glucose and the formation of ATP, which is very necessary for the movement (motility) of sperm. Furthermore, Kasai et al. (41) reported a strong association between mitochondrial membrane activity and sperm velocity parameters. The findings in this study also report the same thing, in which the percentage of sperm velocity parameters is low in the PF bulls and correlates with mitochondrial membranes, sperm motility, and fertility rate. However, sperm velocity is a parameter of sperm movement. It includes part of sperm motility that requires ATP produced in the mitochondria of cells to carry out its normal functions, especially in reaching the site of fertilization in the female reproductive tract.

HSP-70 in sperm, apart from being found on the surface of the membrane, this molecule is also found during the process of capacitation and acrosome reaction, primarily spread in the acrosome and post-acrosome areas of sperm. Rio et al. (42) stated that the presence of this HSP-70 molecule in the acrosome also plays an essential role in helping stabilize sperm plasma membrane proteins which are very important in the fertilization process. This work and prior research reveal that HSP-70 in sperm, primarily in the acrosome, is essential to sperm fertility. According to Khawar et al. (43), the acrosome is a one-of-a-kind membrane organelle positioned in the front region of the sperm nucleus. It plays a role in the sperm penetrating the zona pellucida during fertilization. However, failure of the acrosome reaction due to damage to the acrosome cap of sperm is one of the crucial causes of infertility in bulls (44).

Another sperm quality parameter reported to be affected by the cryopreservation process was the integrity of sperm DNA. Damaged sperm DNA is associated with excess ROS production due to cold shock during sperm cryopreservation (4). Oxidative stress, due to an imbalance in ROS production, can cause DNA damage and affect male fertility. Increased ROS levels injure sperm at the molecular scale by inducing lipid peroxidation of the sperm membrane, impairing protein function, and the incidence of DNA damage, impairing sperm features (7). The findings in this study follow the hypothesis of previous studies. The theory regarding the cause of the DNA damage is also related to the mechanism of HSP-70, as has been studied so far, and is proven in the findings of this research. The gene expression level and protein abundance of HSP-70 are associated with DNA damage. However, overall, every sperm quality parameter tested in this study is related to a fertility rate. This finding is, as previously reported, that sperm quality will affect fertility in the field (3, 45).

Furthermore, our study also showed that PF bulls had lower sperm quality, gene expression levels, and protein abundance of HSP-70. The mechanism may be that the presence of low HSP-70 molecules is not enough to control the increase in oxidative stress, which results in increased lipid peroxidation and the formation of ROS due to a series of cryopreservation processes that will disrupt some of the normal functions of sperm. Wang et al. (46) previously reported on the potential expression level of HSP-90 protein as a reliable and simple marker in predicting freezing resistance in bull sperm. Our study adds the latest information regarding the potential of another variant of HSPs, namely HSP-70, which has great potential as a cryo-tolerance marker in bull sperm and is also associated with various parameters of sperm quality and fertility rate in the field. Even so, various more in-depth studies, primarily related to the HSP-70 mechanism at several freezing stages and in multiple seasons or quite tense weather, will further enrich this study.

## Data availability statement

The original contributions presented in the study are included in the article/supplementary material, further inquiries can be directed to the corresponding authors.

## Author contributions

BeP, BaP, and AK conceptualized and designed the study. BeP and AK curated the data. BeP carried out the investigations and analyzed and interpreted the data. BeP wrote the original draft. BeP, BaP, AK, and MP reviewed and edited the manuscript. All authors contributed to the article and approved the submitted version.

## Conflict of interest

The authors declare that the research was conducted without any commercial or financial relationships that could be construed as a potential conflict of interest.

## Publisher’s note

All claims expressed in this article are solely those of the authors and do not necessarily represent those of their affiliated organizations, or those of the publisher, the editors and the reviewers. Any product that may be evaluated in this article, or claim that may be made by its manufacturer, is not guaranteed or endorsed by the publisher.
